# Supplementary material for: Bioaccumulation of Lead, Cadmium, and Arsenic in a Mining Area and Its Associated Health Effects
Source: Toxics. 2023 Jun 9;11(6):519. doi: 10.3390/toxics11060519 (PMC10301356; doi:10.3390/toxics11060519)
Supplement: Supplementary file 1 [file toxics-11-00519-s001.zip › toxics-2365117-supplementary.pdf]

Table S1. General characteristics of the study participants

	Total	Men	Women	p-value*
Total, n (%)	58 (100)	19 (32.8)	39 (67.2)	
Age, AM±SD (year)	69.4±11.9	67.9±10.2	70.1±12.7	0.513
BMI, AM±SD (kg/m^2)	23.8±3.9	23.7±3.5	23.9±4.1	0.810
Duration of residence, AM±SD (year)	45.3±20.0	49.4±21.1	43.2±19.4	0.272
Mining work experience, n (%)	2 (3.5)	1 (5.3)	1 (2.6)	0.279
Current or ex-smokers, n (%)	12 (20.7)	12 (63.2)	-	<0.001
Alcoholic drinkers, n (%)	28 (48.3)	16 (84.2)	12 (30.8)	<0.001
Rice consumption >50%, n (%)[a]	40 (69.0)	15 (78.9)	25 (64.1)	0.251
Hypertension, n (%)	25 (44.6)	7 (38.9)	18 (47.4)	0.551
Diabetes, n (%)	9 (15.5)	4 (21.1)	5 (12.8)	0.416
Blood Pb, GM (95% CI) (μg/dL)	1.80 (1.54, 2.09)	1.66 (1.25, 2.21)	1.87 (1.55, 2.25)	0.489
Blood Cd, GM (95% CI) (μg/L)	2.12 (1.73-2.59)	1.21 (0.99-1.50)	2.77 (2.17-3.54)	<0.001
Urinary Cd, GM (95% CI) (μg/L)	2.69 (2.23-3.25)	2.02 (1.53-2.66)	3.09 (2.43-3.93)	0.022
Urinay iAs, GM (95% CI) (μg/L)	0.22 (0.14, 0.33)	0.27 (0.11, 0.62)	0.20 (0.12, 0.32)	0.519
Urinary total As, GM (95% CI) (μg/L)	51.76 (42.42, 63.16)	48.75 (36.26, 65.54)	53.30 (40.81, 69.61)	0.706

[Abbreviation] AM arithmetic mean; SD standard deviation; Pb lead; Cd cadmium; iAs inorganic arsenic; total As sum of $As3^+$, $As5^+$, MMA, DMA; GM geometric mean; CI confidence interval;

* *p*-values were calculated by chi-square tests or Wilcoxon tests for the difference between men and women.

[a] Proportions of locally produces rice consumption to that of total rice consumption;

Table S2. Multiple and logistic regression analyses

Factors	Multiple regression		Logistic regression			
	eGFR (ml/min/1.73m^2)		eGFR <90 ml/min/1.73m^2			
	ß	*p*-value	OR	95% Confidence interval		*p*-value
Age (year)	-0.502	0.003	1.16	(1.03	1.31)	0.015
Gender	-0.717	0.858	0.42	(0.03	6.23)	0.532
Duration of residence (year)	-0.091	0.317	0.99	(0.93	1.04)	0.604
Blood Pb (μg/dL)	2.867	0.454	0.96	(0.15	5.97)	0.964
Blood Cd (μg/L)	-5.473	0.142	1.58	(0.21	11.73)	0.650
Urinary Cd (μg/L)	4.368	0.178	0.96	(0.21	4.47)	0.962
Urinary total As (μg/L)	-4.536	0.094	3.86	(0.89	16.65)	0.07

[Abbreviation] eGFR: estimated glomerular filtration rate; OR: Odds Ratio

[Note] The concentrations of blood Pb and Cd, urinary Cd and total As were log-transformed.
